# Non-animal endoscopic training models are also effective for simulation of endoscopic submucosal dissection with adaptive traction strategy

**DOI:** 10.1055/a-2134-8567

**Published:** 2023-08-21

**Authors:** Elena De Cristofaro, Pierre Lafeuille, Jérémie Jacques, Louis-Jean Masgnaux, Timothée Wallenhorst, Clara Yzet, Mathieu Pioche

**Affiliations:** 1 Gastroenterology Unit, Department of Systems Medicine, University of Rome Tor Vergata, Rome, Italy; 2 Gastroenterology and Endoscopy Unit, Edouard Herriot Hospital, Hospices Civils de Lyon, Lyon, France; 3 Gastroenterology and Endoscopy Unit, Dupuytren University Hospital, Limoges, France; 4 Department of Gastroenterology, Pontchaillou University Hospital, Rennes, France; 5 Department of Gastroenterology, Amiens University Hospital, Amiens, France

EndoGel (Fujifilm, Tokyo, Japan) is a new non-animal training model for endoscopic submucosal dissection (ESD) that seems a good option for initial training. The European Society of Gastrointestinal Endoscopy developed an ESD curriculum recommending at least 20 simulated ESD procedures on animal and/or ex vivo models before human practice. However, this training strategy creates ethical, ecological, and economical challenges (e. g. need for dedicated scopes, devices, and rooms). Additionally, there is a low but non-negligible risk of cross contamination.

EndoGel is made with nonbiological materials presented in a cardboard box and simulates the texture of human tissue in these kinds of procedures to help provide a life-like simulation ( Fig. 1 ) without any animal use. The users can perform all steps of the conventional ESD procedure.

**Fig. 1:**
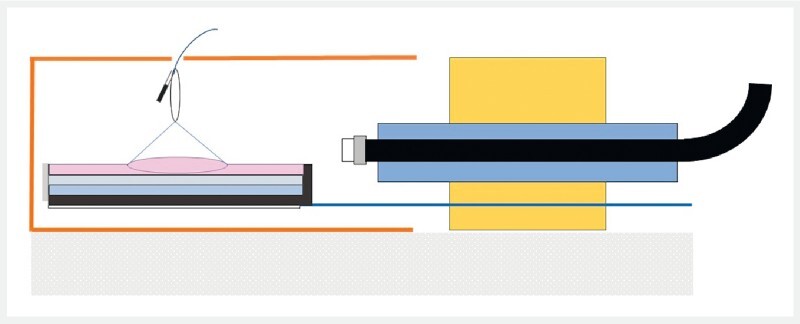
Schematic representation of the EndoGel device (Fujifilm, Tokyo, Japan) with the adaptive traction system.

In recent years, traction-assisted ESD has become the reference method as the traction device facilitates the procedure and reduces the procedure time. However, training in the use of the traction device is also essential for ensuring accurate placement of the device elements. We report the use of EndoGel to perform ESD with an adaptive traction system (A-TRACT 4).

After marking and submucosa injection, circumferential incision and trimming were performed. The four loops of A-TRACT 4 were fixed on lateral edges by clips. The rubber band was fixed to a loop hanging through the top of the cardboard box ( Fig. 1 ) to create 90° traction ( Video 1 ). Submucosal dissection was performed, with optimal submucosal exposure ensured by tightening the A-TRACT 4.

**Video 1** EndoGel device for training in traction-assisted endoscopic submucosal dissection.


The procedure was performed by experts who confirmed good reproducibility and analogy with the real-life experience.

We hypothesize that such a dedicated model, which is ecologically acceptable with no animal components, could facilitate ESD training with adaptive traction strategies. The model allows human scopes to be used in nondedicated rooms without the risk of scope contamination with animal tissue.

Endoscopy_UCTN_Code_TTT_1AU_2AB

## Notes

Endoscopy E-Videos

https://eref.thieme.de/e-videos
*E-Videos* is an open access online section of the journal *Endoscopy* , reporting on interesting cases and new techniques in gastroenterological endoscopy. All papers include a high-quality video and are published with a Creative Commons CC-BY license. Endoscopy E-Videos qualify for HINARI discounts and waivers and eligibility is automatically checked during the submission process. We grant 100% waivers to articles whose corresponding authors are based in Group A countries and 50% waivers to those who are based in Group B countries as classified by Research4Life (see: https://www.research4life.org/access/eligibility/ ). This section has its own submission website at https://mc.manuscriptcentral.com/e-videos

